# Effects of daily glucose fluctuations on the healing response to everolimus-eluting stent implantation as assessed using continuous glucose monitoring and optical coherence tomography

**DOI:** 10.1186/s12933-016-0395-4

**Published:** 2016-05-21

**Authors:** Masaru Kuroda, Toshiro Shinke, Hiromasa Otake, Daisuke Sugiyama, Tomofumi Takaya, Hachidai Takahashi, Daisuke Terashita, Kenzo Uzu, Natsuko Tahara, Daiji Kashiwagi, Koji Kuroda, Yuto Shinkura, Yoshinori Nagasawa, Kazuhiko Sakaguchi, Yushi Hirota, Wataru Ogawa, Ken-ichi Hirata

**Affiliations:** Division of Cardiovascular Medicine, Department of Internal Medicine, Kobe University Graduate School of Medicine, 7-5-1 Kusunoki-cho, Chuo-Ku, Kobe, Hyogo 650-0017 Japan; Department of Preventive Medicine and Public Health, School of Medicine, Keio University, 35 Shinanomachi, Shinjuku-ku, Tokyo, 160-8582 Japan; Division of Diabetes and Metabolism, Department of Internal Medicine, Kobe University Graduate School of Medicine, 7-5-1 Kusunoki-cho, Chuo-Ku, Kobe, Hyogo 650-0017 Japan

**Keywords:** Glucose fluctuation, Continuous glucose monitoring, Mean amplitude of glycemic excursion, Optical coherence tomography

## Abstract

**Background:**

Several studies have revealed that glucose fluctuations provoke oxidative stress that leads to endothelial cell dysfunction, progression of coronary atherosclerosis, and plaque vulnerability. However, little is known regarding their effect on neointimal growth after stenting in patients with coronary artery disease (CAD). We aimed to investigate the effects of glucose fluctuations on neointimal growth after everolimus-eluting stent (EES) implantation.

**Methods:**

This study examined 50 patients who underwent a 9-month follow-up using optical coherence tomography (OCT) after EES implantation. Glucose fluctuation was expressed as the mean amplitude of glycemic excursion (MAGE), and was determined via continuous glucose monitoring before stenting. At the OCT follow-up, we evaluated the percentage of uncovered struts and three-dimensional uniformity of neointimal distribution by calculating the mean neointimal thickness (NIT) within 360 equally-spaced radial sectors for every 1-mm cross-sectional OCT analysis, and assessed the incidence of major adverse cardiovascular events (MACE).

**Results:**

We evaluated 60 lesions in 50 patients. Linear mixed effect models were used to explore the influence of different variables on variability in NIT and the percentage of uncovered struts and to adjust for covariates. Univariate analysis showed that MAGE was most strongly correlated with the previously mentioned OCT measurements (coefficient β ± standard error = 0.267 ± 0.073 and 0.016 ± 0.003, t = 3.668 and 6.092, both P < 0.001, respectively). In multivariate analysis, MAGE had the strongest effect on variability in NIT (coefficient β ± standard error = 0.239 ± 0.093, P = 0.014) and the percentage of uncovered struts (coefficient β ± standard error = 0.019 ± 0.004, P < 0.001). Five lesions in four patients required target lesion revascularization (10.0 %) at a mean duration of 9 months after EES implantation. Compared to non-MACE cases, cases of MACE exhibited a significantly higher MAGE (99 vs. 68; P = 0.004), maximum NIT (580 vs. 330 µm; P = 0.002), and variability in NIT (100 vs. 65; P = 0.007), although there was no significant difference in these groups’ HbA1c levels.

**Conclusions:**

Glucose fluctuation may affect vessel healing after EES implantation in patients with CAD who are receiving lipid-lowering therapy. Therefore, glucose fluctuations may be an important target for secondary prevention after coronary stenting, which is independent of dyslipidemia control.

## Background

Dyslipidemia, and especially high levels of low-density lipoprotein (LDL) cholesterol, has been recognized as one of the most important promoters of late-phase stent restenosis. A large number of clinical trials have reported the beneficial effects of statins for secondary prevention and improved all-cause mortality, as well as for lowering LDL cholesterol levels [1, 2]. However, the limited ability of risk reduction associated with lipid-lowering therapy alone has attracted attention to the unmet need for residual clinical risk management that extends beyond statin use.

Patients with diabetes mellitus (DM) have a particularly high risk of restenosis and target lesion revascularization (TLR), compared to patients without DM [3, 4]. One of the possible mechanisms for this increased risk was thought to be diffuse and accelerated neointimal proliferation within the stented segment [5]. Although the introduction of second-generation drug-eluting stents (DES), such as the everolimus-eluting stent (EES), has markedly reduced the incidence of early- and late-phase stent restenosis, the presence of DM is still associated with an increased risk of restenosis and poor clinical outcomes after percutaneous coronary intervention (PCI) [6, 7]. However, the detailed vascular responses to EES implantation among patients with impaired glucose metabolism has not been fully elucidated.

Sustained hyperglycemia is the underline condition in patients with DM, especially in its advanced stage. Recent studies have revealed that, not only continuous hyperglycemia, but also large glucose fluctuations, such as postprandial hyperglycemia, should be a deleterious factor that drive cardiovascular disease [8–11]. In-vitro analysis have shown that glucose fluctuations may exhibit a more specific triggering effect on oxidative stress and have adverse effects on human endothelial cells [8, 12]. The recent emergence of continuous glucose monitoring (CGM) systems has made it possible to evaluate daily glucose fluctuations in clinical practice. Although insulin resistance might affect neointimal tissue proliferation after 2nd-generation DES implantation, it remains unclear whether glucose fluctuations may affect vessel healing after stent deployment [13].

Numerous reports have described optical coherence tomography (OCT) as a high-resolution intravascular imaging modality that enables detailed assessments of the neointimal proliferation after stenting and plaque character [14, 15]. The present study aimed to investigate the relationship between glucose fluctuations and the arterial response after stenting, which we analyzed using CGM and OCT, respectively.

## Methods

### Patient population

**Fig. 1:**
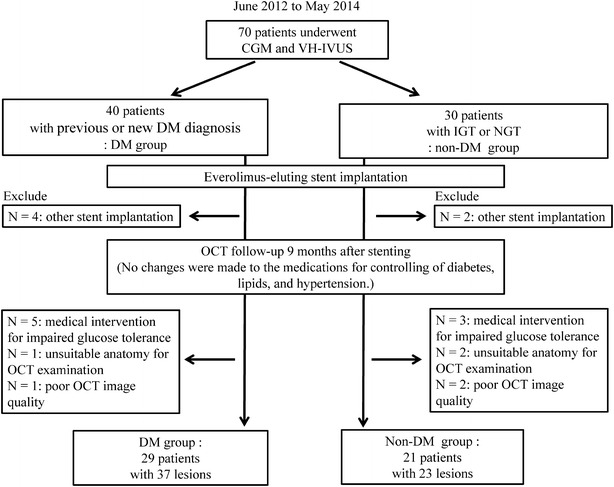
Study flow chart. We enrolled 50 patients who were treated using everolimus-eluting stent [37 lesions in 29 patients with diabetes mellitus (DM) and 23 lesions in 21 patients without DM] and 23 lesions in 21 patients without DM). PCI: percutaneous coronary intervention; CGM: continuous glucose monitoring; VH-IVUS: virtual histology intravascular ultrasound; IGT: impaired glucose tolerance; NGT: normal glucose tolerance; OCT: optical coherence tomography

A previous study [16] enrolled 70 consecutive patients who had undergone PCI using a drug-eluting stent for CAD between June 2012 and May 2014. These patients’ LDL cholesterol levels were <120 mg/dL under statin treatment, or <100 mg/dL under other treatments for dyslipidemia, which included lifestyle management. For the present study, we evaluate only patients who were treated using EES. The exclusion criteria for the present study were: (1) treatment with any other kind of stent; (2) unsuitable anatomy for OCT analysis in the stented segment; (3) severe renal dysfunction; (4) any change in the interventions for controlling diabetes, lipids, and hypertension during the study period; and (5) the patients with any antidiabetic medications other than life-style management after the CGM. In total, we enrolled 50 patients who treated with EES and were followed-up after 9 months via OCT (Fig. 1). In addition, we divided the patients into groups of patients with and without DM.

This study was approved by the ethics committee of Kobe University, and was performed in accordance with the tenets of the Declaration of Helsinki. All enrolled patients provided their written informed consent for enrollment in the study.

### Study protocol

At the index stent procedure, a fasting blood sample was obtained and submitted for testing to evaluate the levels of creatinine, glycosylated hemoglobin (HbA1c), LDL cholesterol, high-density lipoprotein (HDL) cholesterol, and triglycerides. In addition, a 75-g oral glucose tolerance test was performed for all patients, and the levels of plasma glucose and immunoreactive insulin were evaluated immediately before and 120 min after the oral glucose load. Subcutaneous interstitial glucose levels were monitored over a 3-day period using the CGM System iPro™2 (Medtronic, Northridge, CA, USA).

**Fig. 2:**
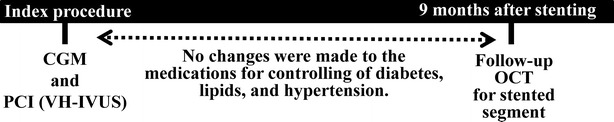
Study protocol. At the index procedure, patients underwent continuous glucose monitoring (CGM) and percutaneous coronary intervention (PCI) that was guided via virtual histology intravascular ultrasound (VH-IVUS). We performed follow-up optical coherence tomography (OCT) at 9 months after stenting. No changes were made during the study to any of the medications for controlling diabetes, lipids, and hypertension

After the CGM examination, all patients underwent a catheterization procedure for PCI in the native coronary arteries, which was guided by intravascular ultrasound (IVUS) (Eagle Eye^®^ Platinum 3.5F 20-MHz; Volcano Corp, Rancho Cordova, CA, USA) and were treated using EES implantation. The IVUS procedure was performed in a standard manner, which used an automated motorized 0.5 mm/s pullback. Culprit lesions were identified by analyzing the pre-crisis and inter-crisis electrocardiograms, left ventricular wall motion abnormalities, and angiographic lesion appearances. At 9 months after the index stent procedure, we performed the follow-up coronary angiography and OCT examinations of the stented segment, and evaluated the incidence of major adverse cardiovascular events (MACE) (Fig. 2).

All patients were advised to take dual antiplatelet therapy, which consisted of acetylsalicylic acid (100 mg/day) and clopidogrel (75 mg/day) for at least 12 months after EES implantation. However, there were no changes in the treatments for controlling diabetes, lipids, and hypertension before the 9-month follow-up.

### Continuous glucose monitoring and analysis of glucose fluctuations

The CGM system has been described in the previous study [16]. In brief, CGM was performed for 3 consecutive days before PCI, and the median variables on days 2 and 3 [24-h mean glucose levels, time in hyper/hypoglycemia, and mean amplitude of glycemic excursion (MAGE)] were calculated using CGM analysis software (CareLink iPro; Medtronic, Northridge, CA, USA). In this context, Service et al. [17] have proposed that MAGE represents the fluctuations in blood glucose levels over a 24-h period, and MAGE was calculated in the present study using the daily variations in blood glucose levels (recording using CGM over a 2-day period). All patients received optimal meals during the CGM (25–28 kcal/kg of ideal body weight; 60 % carbohydrates, 15–20 % protein, and 20–25 % fat).

### Pre-intervention IVUS and VH-IVUS analysis

At the index PCI procedure, IVUS was used to measure a segment of the target vessel that had a minimum length of 30 mm and extended from the distal side of the target lesion to the coronary ostium. The manual contour detection in both the lumen and the media adventitia interface was performed by two experienced analysts who were blinded to baseline clinical and angiographic lesion characteristics. We analyzed the whole lesion volume, and then calculated the volumes of the lumen, vessel, and plaque (vessel minus lumen) using Simpson’s method. VH-IVUS automatically classified the plaque into four major components [fibrous (labeled green), fibro-fatty (labeled greenish-yellow), necrotic core (NC; labeled red), and dense calcium (labeled white)] [18]. The ratio of each plaque component in the culprit lesions was expressed as a percentage of the total plaque volume.

### OCT examination

The frequency-domain OCT examination was performed at 9 months after stenting, as previously reported [14]. In brief, a 0.014-in standard guide wire was positioned distally in the target vessel, and the frequency-domain OCT catheter (C7 or C8 Dragonfly™; St. Jude Medical, St. Paul, MN, USA) was advanced to the distal end of the target lesion. The entire region of interest was scanned using the integrated automated pullback device (20 mm/s). For image acquisition, blood in the coronary artery was replaced with iodine contrast media that was continuously flushed using a power injector, which creates an essentially blood-free environment. The volume and infusion flow rates were decided by the operator and ranged from 8 to 20 cm^3^ at 3–7 cm^3^/s and 400 psi.

### OCT analysis

The off-line OCT analysis was performed using dedicated software (LightLab Imaging Inc, Westford, MA, USA), and all images were analyzed by independent observers who were blinded to the clinical presentation and lesion characteristics. For the quantitative analysis, cross-sectional OCT images were analyzed at 1-mm intervals. Bifurcation cross-sections with side branches were excluded from this analysis. Neointimal thickness (NIT) inside each stent strut and stent area was measured. The stent and lumen areas were manually measured and the neointima area was calculated as the stent area minus the lumen area. Struts with an NIT of 0 μm were defined as uncovered struts. The frequency of uncovered struts was calculated as the number of uncovered struts divided by the total number of stent struts. To evaluate asymmetrical stent expansion, a stent eccentricity index (SEI) was defined as the minimum stent diameter divided by the maximum stent diameter in each cross-section, and the average and minimum SEIs were calculated for each stent [19]. It can be assumed that vessel healing after DES implantation comprise of the amount of neointimal growth leading to restenosis and uncovered strut which could be a substrate of thrombosis [20, 21]. Therefore, we defined vessel healing as the conditions of neointimal growth and uncovered struts after stenting. Intracoronary thrombus was defined as an irregular mass protruding beyond the stent strut into the lumen, with significant attenuation behind the mass [22]. The presence of in-stent thrombi required the agreement of two independent experienced observers, which provided acceptable intra- and inter-observer agreement (intra-observer, kappa = 0.9; inter-observer, kappa = 0.815).

### Three-dimensional assessment of neointimal distribution

**Fig. 3:**
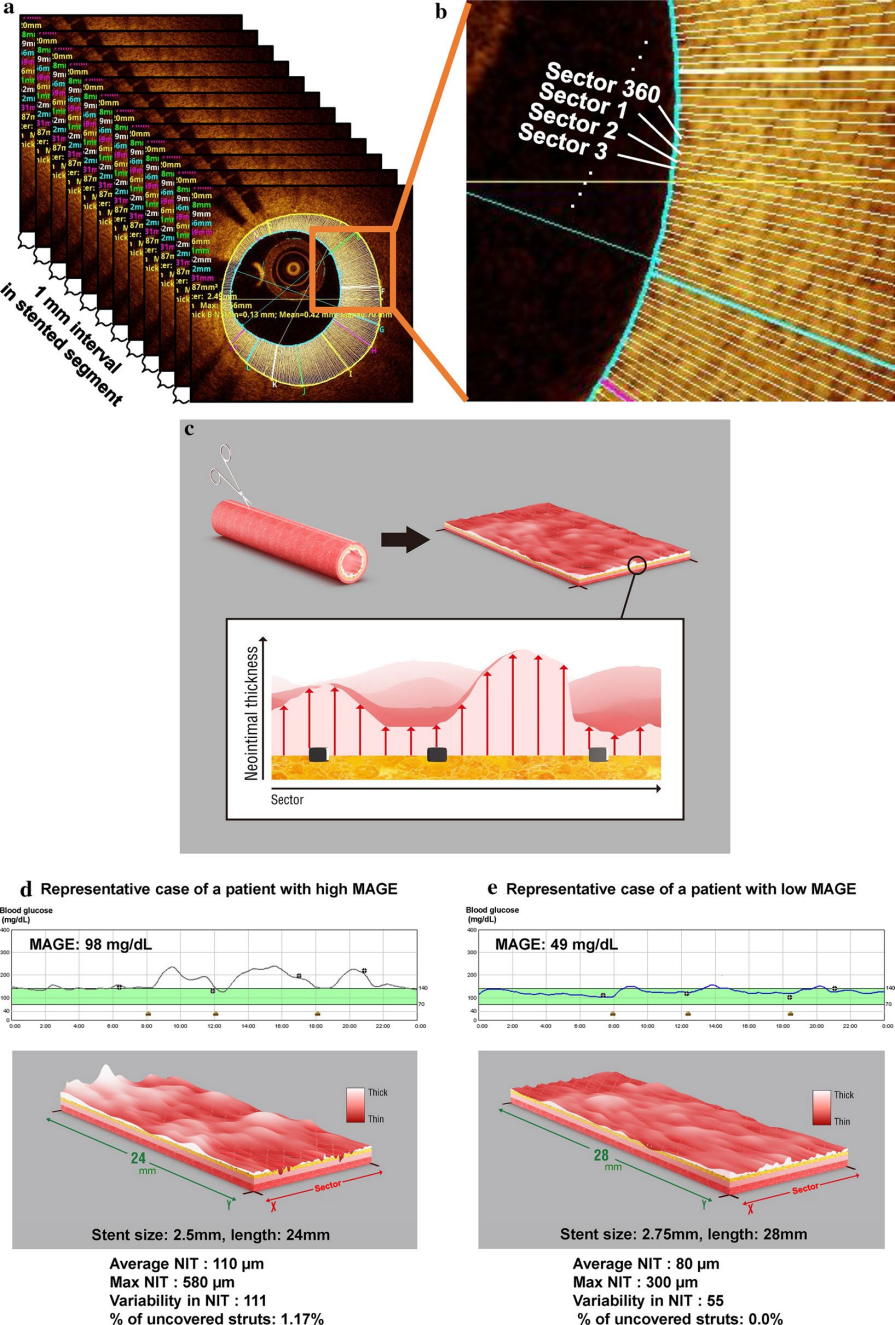
Three-dimensional assessment of the neointimal distribution. **a** The lumen and stent areas were manually traced every 1 mm. **b** The neointimal area was divided into 360 circumferential sectors (each section is 1°, which is referenced from the lumen’s center). The mean neointimal thickness (NIT) was calculated for each sector in each 1-mm section, and the same sector’s thicknesses were compared throughout the entire stented segment. **c** To visualize the two-dimensional layout, the stent surface was cut along the longitudinal direction and flattened into a *rectangular shape*. The roughness of the neointima (variability in NIT) within the whole stent was evaluated based on the standard deviation (SD) of the NITs from the same 1°-sector along the entire stented segment. Representative cases are shown for a patient with high mean amplitude of glycemic excursion (MAGE) (**d**) and a patient with low MAGE (**e**)

The three-dimensional uniformity of neointima distribution within the stented segment was evaluated to investigate the association between the variability in neointima proliferation and glucose variability [23]. First, the lumen and stent contours on the cross-sectional images were manually traced at 1-mm intervals. Next, the software divided the neointima area (between the stent and lumen) into 360 equally spaced circumferential sectors (each sector spanned 1°) for each cross-sectional OCT image (Fig. 3). In this process, the lumen’s center was used as the reference point for sector division. The mean NIT in each sector was automatically calculated, and the roughness of the neointima (variability in NIT) within the whole stent was evaluated based on the standard deviation (SD) of the NITs from the same 1°-sector along the entire stented segment. Both quantitative coronary analysis by coronary angiography (CAG) and IVUS are robust tools for the quantification of neointimal tissue, so the resolution of CAG and IVUS (i.e., 100–150 μm) may not be sufficient for detecting a suppressed, small degree of neointimal hyperplasia after DES implantation. OCT has a higher resolution (10–15 μm) and can clearly visualize thin neointima and other subtle changes in vascular response in more detail; therefore, we used an OCT-based 3-D method to quantitatively evaluate the variability in neointimal distribution after stenting to investigate the uniformity of neointima proliferation.

### Outcome variables and definitions

Clinical outcome data (mean follow-up: 277 days) were obtained by reviewing outpatient records and via telephone interviews. The primary outcomes were death, myocardial infarction (MI), stent thrombosis according to the ARC definition [24], clinically-driven TLR, and MACE (cardiac death, MI, and TLR) at the 9-month follow-up. All deaths were considered cardiac-related unless an unequivocal non-cardiac cause was established, and all events were carefully verified by independent clinicians.

### Statistical analysis

All data were presented as mean ± standard deviation or proportions. Variables were compared using Fisher’s exact test and Student’s t test, as appropriate. Linear mixed effect models were used to explore the influence of different variables on variability in NIT and the percentage of uncovered struts and to adjust for covariates. Univariabale analysis was first performed, and all the variables that satisfied P < 0.1 were entered en bloc in the multivariable model, along with age and sex as background variables. To assess the inter- and intra-observer variabilities, the results were compared using the kappa test of concordance for categorical data, and a Bland–Altman plot was used for continuous data. All analyses were performed using SPSS software (version 22; SPSS Inc., Chicago, IL, USA), and differences with a P value of < 0.05 were considered statistically significant.

## Results

### Baseline patient characteristics

We enrolled 50 patients (Fig. 1), and their baseline characteristics are shown in Table 1. There were no significant differences between the DM and non-DM groups, except for in their levels of HbA1c, 1,5 anhydroglucitol, and glycoalbumin. Both groups exhibited similar medications at admission, with the exception of anti-diabetes treatment. However, the DM group typically exhibited significantly greater CGM-related values, compared to the non-DM group. There were no significant differences in the minimum blood glucose levels and time in hypoglycemia.

**Table 1:** Baseline patient characteristics

	Overall (n = 50)	DM (n = 29)	Non-DM (n = 21)	P value (DM vs. non-DM)
Age (years)	70.1 ± 10.3	71.6 ± 8.8	68.0 ± 12.1	0.23
BMI (kg/m^2^)	24.2 ± 3.4	24.5 ± 3.0	23.8 ± 4.0	0.45
Male	43 (86.0)	24 (82.8)	19 (90.5)	0.36
DM	29 (58.0)	29 (100)	0 (0.0)	–
Hypertension	37 (74.0)	22 (75.9)	15 (71.4)	0.72
Dyslipidemia	44 (88.0)	27 (93.1)	17 (81.0)	0.19
Smoking	36 (72.0)	21 (72.4)	15 (71.4)	0.38
Current	10 (20.0)	4 (13.8)	6 (28.6)	
Former (quit >3 months)	26 (52.0)	17 (58.6)	9 (42.9)	
Prior myocardial infarction	12 (24.0)	6 (20.7)	6 (28.6)	0.52
Prior PCI	25 (50.0)	16 (55.2)	9 (42.9)	0.39
Systolic blood pressure (mmHg)	122.1 ± 11.8	122.1 ± 11.3	122.1 ± 12.7	0.99
Diastolic blood pressure (mmHg)	63.3 ± 6.9	62.0 ± 6.5	65.2 ± 7.2	0.10
Left ventricular ejection fraction (%)	59.3 ± 10.2	61.7 ± 7.2	55.7 ± 13.0	0.082
Duration of DM (years)	3.4 ± 6.6	7.9 ± 1.5	–	–
HbA1c (NGSP) (%)	6.4 ± 0.9	6.8 ± 1.0	5.8 ± 0.3	<0.001
1,5-AG (μg/mL)	15.7 ± 7.7	13.2 ± 6.7	19.5 ± 7.6	0.004
Glycoalbumin (%)	16.5 ± 3.2	17.6 ± 3.5	14.8 ± 1.8	0.001
75-g OGTT				
Fasting PG (mg/dL)	102 ± 21	111 ± 23	89 ± 8	<0.001
2-h PG (mg/dL)	202 ± 79	249 ± 70	139 ± 33	<0.001
Fasting IRI (μU/mL)	7.4 ± 6.0	7.9 ± 7.2	6.7 ± 4.0	0.49
2-h IRI (μU/mL)	103 ± 106	111 ± 118	91 ± 86	0.53
HOMA R	2.0 ± 2.4	2.4 ± 3.1	1.5 ± 0.8	0.21
HOMA β	80.4 ± 59.7	63.6 ± 40.5	102.7 ± 73.6	0.036
Total cholesterol (mg/dL)	157.0 ± 25.9	157.1 ± 23.6	156.8 ± 29.4	0.96
LDL cholesterol (mg/dL)	88.6 ± 18.1	90.2 ± 17.6	86.2 ± 18.9	0.44
HDL cholesterol (mg/dL)	45.8 ± 12.1	42.6 ± 10.0	50.1 ± 13.6	0.039
Triglyceride (mg/dL)	133.2 ± 53.5	152.55 ± 53.5	107.1 ± 38.6	0.002
CRP (mg/dL)	0.17 ± 0.28	0.14 ± 0.21	0.20 ± 0.34	0.47
Creatinine (mg/dL)	0.98 ± 0.25	0.99 ± 0.24	0.96 ± 0.26	0.63
Medications during the study				
Aspirin	42 (84.0)	25 (86.2)	17 (81.0)	0.45
Thienopyridine	23 (46.0)	13 (44.8)	10 (47.6)	0.85
Statin	38 (76.0)	24 (82.8)	14 (66.7)	0.19
EPA	4 (8.0)	2 (6.9)	2 (9.5)	0.56
Ezetimibe	4 (8.0)	2 (6.9)	2 (9.5)	0.56
Fibrate	1 (2.0)	0 (0.0)	1 (4.8)	0.42
ACE-I/ARB	27 (54.0)	15 (51.7)	12 (57.1)	0.70
Beta-blocker	17 (34.0)	10 (34.5)	7 (33.3)	0.93
Insulin	0 (0.0)			
Metformin	2 (4.0)	2 (6.9)	0 (0.0)	0.33
SU	7 (14.0)	7 (24.1)	0 (0.0)	0.016
α-GI	3 (6.0)	3 (10.3)	0 (0.0)	0.19
DPP4-I	9 (18.0)	9 (18.0)	0 (0.0)	0.004
Continuous glucose monitoring variables				
MAGE (mg/dL)	71 ± 33	80 ± 35	58 ± 28	0.018
Mean BG (mg/dL)	128 ± 21	137 ± 24	116 ± 9	<0.001
Max BG (mg/dL)	210 ± 42	226 ± 44	188 ± 27	<0.001
Min BG (mg/dL)	72 ± 23	75 ± 27	70 ± 16	0.62
Time in hyperglycemia (h)	23.2 ± 24.3	32.9 ± 27.4	9.1 ± 5.6	<0.001
Time in hypoglycemia (h)	1.7 ± 3.0	1.9 ± 3.5	1.5 ± 2.2	0.62

Values are mean ± standard deviation or number (%). Time in hyperglycemia and hypoglycemia were defined as the time when blood glucose levels were >140 mg/dL and <70 mg/dL, respectively

*1,5-AG* 1,5 anhydroglucitol, *75* *g OGTT* 75-g oral glucose tolerance test, *α-GI* α-glucosidase inhibitor, *ACE-I* angiotensin converting enzyme-inhibitor, *ARB* angiotensin II receptor blocker, *BG* blood glucose, *BMI* body mass index, *CRP* C-reactive protein, *DM* diabetes mellitus, *DPP4-I* dipeptidyl peptidase-4 inhibitor, *EPA* eicosapentaenoic acid, *HbA1c* glycated hemoglobin, *HDL cholesterol* high-density lipoprotein cholesterol, *HOMA β* homeostasis model assessment beta, *HOMA R* homeostasis model assessment ratio, *IRI* immunoreactive insulin, *LDL* *cholesterol* low-density lipoprotein cholesterol, *MAG*: mean amplitude of glycemic excursion, *NGSP* National Glycohemoglobin Standardization Program, *PCI* percutaneous coronary intervention, *PG* plasma glucose, *SU* sulfonylureas

### Lesion characteristics

The 50 patients had 60 lesions, which included 37 lesions in 29 patients with DM and 23 lesions in 21 patients without DM. The plaque characteristics that were obtained via VH-IVUS are shown in Table 2. The percentage of necrotic core volume within the plaque was numerically higher in the DM group, compared to that in the non-DM group. Stent length in the DM group was longer than that in the non-DM group.

**Table 2:** Lesion and procedural characteristics

	Overall (n = 60)	DM (n = 37)	Non-DM (n = 23)	P value (DM vs. non-DM)
Lesion location				0.72
LAD	26 (43.3)	15 (40.5)	11 (47.8)	
LCx	15 (25.0)	9 (24.3)	6 (26.1)	
RCA	19 (31.2)	13 (35.1)	6 (26.1)	
AHA/ACC lesion classification				0.27
Type A/B1	12 (20.0)	6 (16.2)	6 (26.1)	
Type B2/C	48 (80.0)	31 (83.8)	17 (73.9)	
Chronic total occlusion	8 (13.3)	6 (16.2)	2 (8.7)	0.34
Bifurcation	16 (26.7)	9 (24.3)	7 (30.4)	0.60
IVUS measurements before stenting				
Plaque volume				
Absolute data (mm^3^)	98.1 ± 67.7	104.0 ± 77.3	89.2 ± 50.7	0.48
Plaque burden (%)	58.1 ± 10.5	57.4 ± 11.7	59.2 ± 8.4	0.62
Lesion length (mm)	14.4 ± 9.1	14..7 ± 10.3	14.0 ± 7.1	0.81
Fibrous				
Absolute data (mm^3^)	35.1 ± 25.8	37.6 ± 29.0	31.4 ± 20.3	0.44
Relative data (%)	60.4 ± 9.2	60.6 ± 10.0	59.9 ± 8.2	0.80
Fibro-fatty				
Absolute data (mm^3^)	7.3 ± 5.5	7.8 ± 6.3	6.5 ± 4.0	0.41
Relative data (%)	12.7 ± 5.2	12.1 ± 3.8	13.6 ± 6.8	0.37
Dense-calcium				
Absolute data (mm^3^)	5.2 ± 5.4	5.5 ± 6.1	4.7 ± 4.2	0.61
Relative data (%)	7.7 ± 6.0	7.4 ± 5.8	8.3 ± 6.5	0.64
Necrotic-core				
Absolute data (mm^3^)	12.9 ± 12.4	14.4 ± 14.1	10.7 ± 9.4	0.34
Relative data (%)	19.1 ± 5.3	19.8 ± 4.6	18.2 ± 6.1	0.33
Procedural characteristics				
Stent length (mm)	27.2 ± 13.7	30.2 ± 15.8	22.3 ± 7.4	0.011
Stent size (mm)	3.0 ± 0.4	2.9 ± 0.4	3.1 ± 0.4	0.14
Maximum inflation pressure (atm)	11.1 ± 1.4	11.2 ± 1.3	11.0 ± 1.6	0.55
Overlap stenting	11 (18.3)	10 (27.0)	1 (4.3)	0.026
Post-dilatation	47 (78.3)	27 (73.0)	20 (87.0)	0.17
Rotablation	6 (10.0)	5 (13.5)	1 (4.3)	0.25

Values are mean ± standard deviation or number (%)

*AHA/ACC* American Heart Association/American College of Cardiology, *IVUS* intravascular ultrasound, *LAD* left anterior descending artery, *LCx* left circumflex artery, *RCA* right coronary artery

### OCT measurements

The 9-month OCT revealed that the DM group exhibited trends toward a smaller mean stent area, minimum stent area, and lumen area. The mean NIT and neointimal area were numerically higher in the DM group, compared to those in the non-DM group (Table 3). In addition, the DM group exhibited a trend towards greater variability in NIT, compared to the non-DM group. The frequency of uncovered struts was similar between the two groups, although the DM group exhibited a marginally higher frequency of thrombus attachment (13.5 vs. 0.0 %, P = 0.08). In addition, patients having lesions with intra-stent thrombus showed significantly higher MAGE than those without thrombus (103 ± 25 vs. 68 ± 34; P = 0.027, respectively).

**Table 3:** Optical coherence tomography measurements

	Overall (n = 60)	DM (n = 37)	Non-DM (n = 23)	P value (DM vs. non-DM)
OCT follow-up duration (days)	277 ± 69	287 ± 77	261 ± 51	0.16
Mean stent area (mm^2^)	6.7 ± 1.9	6.3 ± 1.8	7.2 ± 1.9	0.063
Mean minimum stent area (mm^2^)	5.3 ± 1.8	5.0 ± 1.7	5.8 ± 1.7	0.073
Mean SEI	0.89 ± 0.03	0.90 ± 0.03	0.88 ± 0.02	0.060
Average of minimum SEI	0.79 ± 0.07	0.79 ± 0.07	0.78 ± 0.06	0.52
Mean lumen area (mm^2^)	6.1 ± 1.9	5.7 ± 1.77	6.7 ± 1.9	0.051
Mean number of struts	271 ± 112	292 ± 124	237 ± 83	0.070
Mean number of uncovered struts	2.2 ± 2.9	2.4 ± 3.2	1.9 ± 2.2	0.47
Frequency of uncovered struts (%)	0.78	0.79	0.78	0.97
Mean neointimal thickness (µm)	82 ± 35	86 ± 36	75 ± 33	0.28
Mean neointima area (mm^2^)	0.63 ± 0.31	0.65 ± 0.29	0.61 ± 0.35	0.60
Average of max neointimal thickness (µm)	350 ± 180	420 ± 180	380 ± 150	0.44
Variability in neointima thickness	67 ± 26	73 ± 29	59 ± 18	0.060
Frequency of stent with in-stent thrombi	5 (8.3)	5 (13.5)	0 (0.0)	0.080

Values are mean ± standard deviation or number (%)

*OCT* optical coherence tomography, *SEI* stent eccentricity index

### Associations of OCT measurements with diabetic control markers and non-glycemic metabolic variables

Linear mixed effect models were used to explore the influence of different variables on variability in NIT and the percentage of uncovered struts and to adjust for covariates. Univariable analysis was first performed (Table 4), revealed that MAGE was most strongly correlated with the previously mentioned OCT measurements (coefficient β ± standard error = 0.267 ± 0.073 and 0.016 ± 0.003, t = 3.668 and 6.092, both P < 0.001, respectively). However, none of the other clinical and laboratory data than glycemic variables were correlated with any OCT measurements. All the variables that satisfied P < 0.1 were entered en bloc in the multivariable model, along with age and sex as background variables (Table 5). As a result, the model of variability in NIT was adjusted for age, sex, MAGE, time in hypoglycemia, and HOMA R, and the model of percentage of uncovered struts by age, sex, MAGE, 1,5 AG, and time in hypoglycemia. In the multivariable, a higher MAGE was independently associated with greater variability in NIT and a higher frequency of uncovered stent struts (coefficient β ± standard error = 0.239 ± 0.093 and 0.019 ± 0.004, t = 2.564 and 4.869, P = 0.014 and <0.001, respectively).

**Table 4:** Correlations between optical coherence tomography measurements and various metabolic markers

Variables	Coefficients	SE	t	p value
For variability of NIT				
MAGE	0.267	0.072	3.668	0.0006
Age	−0.071	0.278	−0.258	0.80
HbA1c	0.867	4.257	0.203	0.83
1,5 AG	−0.403	0.382	−1.054	0.29
Mean BG	0.210	0.131	1.596	0.11
Time in hypoglycemia	1.979	0.894	2.214	0.03
DM duration	0.738	0.473	1.560	0.12
HOMA R	−2.247	1.183	−1.899	0.06
Male	−12.36	8.000	−1.545	0.12
LDL cholesterol	0.002	0.152	0.018	0.98
HDL cholesterol	0.243	0.251	0.967	0.33
TG	−0.047	0.052	−0.902	0.37
CRP	−8.176	13.66	−0.598	0.55
For percentage of uncovered struts				
MAGE	0.016	0.002	6.092	<0.0001
Age	0.020	0.011	1.740	0.11
HbA1c	0.183	0.180	1.016	0.31
1,5 AG	−0.028	0.016	−1.760	0.084
Mean BG	0.006	0.005	1.135	0.26
Time in hypoglycemia	0.072	0.039	1.835	0.072
DM duration	−0.018	0.020	−0.889	0.37
HOMA R	−0.067	0.051	−1.306	0.19
Male	0.710	0.334	2.122	0.039
LDL cholesterol	0.002	0.006	0.376	0.70
HDL cholesterol	0.009	0.010	0.850	0.39
TG	−0.001	0.002	−0.810	0.42
CRP	0.794	0.576	1.379	0.17

*BG* blood glucose, *CRP* C-reactive protein, *DM* diabetes mellitus, *HbA1c* glycated hemoglobin, *HDL* cholesterol: high-density lipoprotein cholesterol, *HOMA R* homeostasis model assessment ratio, *LDL* *cholesterol* low-density lipoprotein cholesterol, *MAGE* mean amplitude of glycemic excursion, *TG* triglycerides

**Table 5:** Linear mixed effect model adjusted with confounders. (A) Variability in NIT and (B) Frequency of uncovered stent struts are the dependent variable

Variables	Coefficients	SE	T	P value
Variability in neointimal thickness				
MAGE	0.239	0.093	2.564	0.014
Time in hypoglycemia	0.159	1.060	0.150	0.88
HOMA R	−1.619	1.108	−1.461	0.15
Frequency of uncovered stent struts				
MAGE	0.018	0.004	4.869	<0.0001
1,5 AG	−0.002	0.013	−0.149	0.88
Time in hypoglycemia	−0.057	0.040	−1.405	0.16

*1,5 AG* 1,5 anhydroglucitol, *HOMA R* homeostasis model assessment ratio, *MAGE* mean amplitude of glycemic excursion

### Association between plaque characteristics and follow-up OCT findings

We investigated the association between the underlying plaque characteristics, as obtained via IVUS at the index PCI, and the 9-month OCT findings. A significant correlation was observed between the percentage of necrotic core volume within the plaque and the percentage of uncovered struts (r = 0.415, P = 0.005), although the percentage of necrotic core volume within the plaque was not associated with maximum NIT and variability in NIT.

### Clinical outcomes

**Fig. 4:**
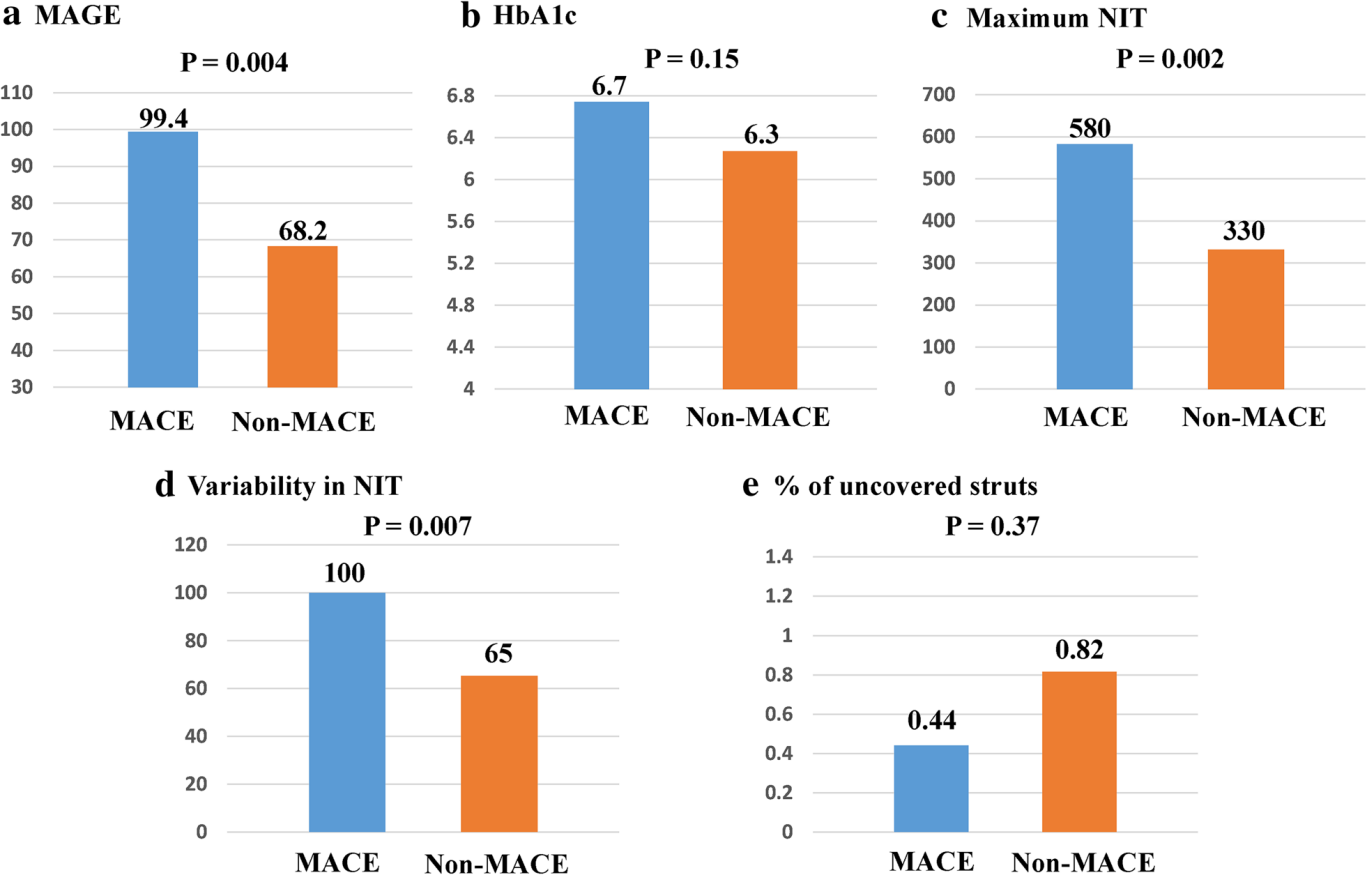
Comparing cases with and without major adverse cardiac events (MACE). The mean amplitude of glycemic excursion in MACE cases was significantly higher than that in non-MACE cases (**a**), whereas the HbA1c levels did not significantly differ between the two groups (**b**). The maximum neointimal thickness (NIT) (**c**) and variability in NIT (**d**) were significantly greater in MACE cases, compared to those in non-MACE cases. There was no significant difference between the two groups in the percentage of uncovered struts (**e**)

The clinical outcomes at 9 months were available for all 50 patients (Table 6), and five lesions in four patients required TLR (10.0 %) at a mean duration of 311 days after EES implantation. There were no cases of cardiac-related death, MI, or stent thrombosis; thus, the incidence of MACE was 10.0 % at 9 months. Compared to non-MACE cases, cases of MACE exhibited a significantly higher MAGE, maximum NIT, and variability in NIT, although there was no significant difference in these groups’ HbA1c levels (Fig. 4).

**Table 6:** Clinical events

	MAGE	HbA1c	Variability in neointimal thickness	% of uncovered struts	MACE
78-year-old man	73	5.9	60	0.6	TLR
77-year-old man	120	7.8	186	0	TLR
71-year-old man	99	6.7	74	0	TLR
71-year-old man	99	6.7	100	0.8	TLR
80-year-old women	101	6.6	79	0.8	TLR
All MACE cases	99 ± 15	6.7 ± 0.7	100 ± 53	0.4 ± 0.4	
Non-MACE cases	68 ± 34	6.4 ± 0.9	65 ± 21	0.8 ± 0.9	

*MACE* major cardiovascular events, *TLR* target lesion revascularization

## Discussion

The present study investigated the effects of glucose fluctuation on vessel healing after second-generation DES implantation in patients with CAD who were receiving dyslipidemia treatments. Our main findings are: (1) among patients with CAD and dyslipidemia management who are referred for PCI, large glucose fluctuations are an independent risk factor for impaired uniform vessel healing after second-generation DES implantation; and (2) compared to non-MACE cases, patients with MACE had a higher incidence of delayed vessel healing, which was accompanied by larger glucose fluctuations.

### The relationship between glucose fluctuation and arterial response after stenting

Despite DES’ efficacy in reducing neointimal proliferation and restenosis, DES failure and restenosis still occur and are more frequent among patients with abnormal glucose torelance [25]. Furthermore, unfavorable neointimal proliferation, as assessed using OCT, is more common among patients with DM [26, 27]. Moreover, diabetes is associated with hormonal and vascular abnormalities that promote smooth muscle cell proliferation after vascular injury, which includes injury after coronary interventions [28]. However, it remains unknown regarding what factors have the greatest effect on vessel reactions to DES in patients with DM. The present study revealed that glucose fluctuations had a stronger positive correlation with distorted neointimal hyperplasia and incomplete neointimal coverage in the stented segment, compared to other glycemic variables (e.g., HbA1c), regardless of DM status. Moreover, multivariate analysis revealed that glucose fluctuation was independently associated with delayed post-stenting vessel healing. In this context, increased smooth muscle proliferation in patients with diabetes might be caused by mitogens (such as platelet-derived growth factor and insulin-like growth factor) that stimulate cell growth, as well as the deleterious effects of endothelial dysfunction and excessive extracellular matrix production [29, 30]. Furthermore, recent studies have suggested that large glucose fluctuations are associated with pro-atherogenic factors, such as oxidative stress, inflammation, and endothelial dysfunction; this relationship was not observed for sustained hyperglycemia that is indicated by HbA1c and fasting plasma glucose [8, 31]. Risso et al. [12] have also explored the effect of fluctuating glucose on endothelial cells, and reported that apoptosis was enhanced in human endothelial cells that were exposed to intermittently high glucose concentrations, rather than prolonged hyperglycemic exposure. On the other hand, Beusekom et al. [32] reported that stent implantation induced endothelial dysfunction in porcine coronary arteries. Moreover, in recent studies, it is speculated that impaired barrier function of the endothelium in the stented segment may allow a greater amount of lipoproteins to enter the sub-endothelial space, leading to the development of abnormal neointimal growth and atherogenic changes [33]. These findings suggest that glucose fluctuations may adversely cause endothelial dysfunction in the stented segment, leading to the development of abnormal neointimal growth and coverage, which supports the findings of the present study.

### Local arterial healing and future clinical events

Previous studies have demonstrated that DM is associated with an increased risk of in-stent restenosis and TLR [3, 4, 34]. As its cause, DM is an inflammatory proliferative disease that may be susceptible to rheological effects, such as the low wall shear stress and high oscillatory shear that are caused by turbulent flow, which is induced by uneven neointimal distribution and may lead to a pro-oxidant, pro-atherosclerotic, and pro-coagulative status [35]. These phenomena might be accelerated by large glucose fluctuations [8, 12], which may explain the heterogeneous neointimal growth that we observed. For example, all five MACE cases exhibited significantly higher MAGE, compared to the non-MACE cases. In addition, the variability in NIT was significantly greater in the MACE cases, compared to the non-MACE cases. Given that the MACE cases only involved TLR, our results indicate that large glucose fluctuations affected the heterogeneous neointimal growth, which may lead to late-phase stent re-stenosis. In addition, uneven neointimal distribution is associated with intra-stent thrombosis after DES implantation [19]. Inhomogeneous neointimal growth coupled with the high incidence of thrombus attachment was observed in the present study. The previous study has shown that yellow plaque detected by angioscopy could be associated with intrastent thrombus formation, and yellow plaque following DES implantation may reflect neoatherosclerotic change in the stented segment, which might be a risk of late stent failure [36]. Taken together, higher variability in NIT after 2nd generation DES implantation might cause higher incidence of intrastent thrombus, leading to future clinical events.

Several observational studies have reported that strict early diabetes control helps prevent macroangiopathy [37], which suggests a need for earlier diagnosis and treatment of diabetes and glucose intolerance. The present study demonstrated that plaque characteristics, which were obtained via pre-intervention IVUS, were not associated with the variability in NIT but with the percentage of uncovered struts after stenting. In our previous studies, a large glucose fluctuation is the independent risk factor for the development of necrotic core within the coronary plaque and formation of TCFA in CAD patients who are under treatment for dyslipidemia [10, 16]. The recent study demonstrated that uncovered, malapposed, and protruding stent struts may be more frequent in vulnerable lesions compared with stable lesions after DES implantation [38]. These findings seem to indicate that glucose fluctuation may promote the formation of unstable plaque, leading to more frequent occurrence of uncovered struts after stent deployment. In addition, glucose variability was significantly and positively correlated with the heterogeneous neointimal growth that might be the precursor to late-phase stent restenosis. Taken together, controlling glucose fluctuations may help avoid heterogeneous neointimal growth and unfavorable vessel healing, leading to prevent future clinical events in patients with coronary stents. Nevertheless, a large-scale prospective study is needed to confirm whether additional glucose fluctuation control can decrease the incidence of late clinical events.

## Limitations

This study had several limitations. First, the single-center design and relatively small sample size may have introduced selection bias. Second, we only assessed glycemic variables using CGM at the index procedure, and excluded any patients with changes in their related medical interventions. However, changes in these interventions might affect glycemic metabolism and the related follow-up findings. Therefore, future studies should examine the effects of changing medical interventions on glucose fluctuation, as these changes may be relevant to the effect of MAGE on vessel healing after stenting. Third, to reduce the influence of the patients’ lipid profiles, we excluded patients whose LDL cholesterol levels were >120 mg/dL under statin treatment or >100 mg/dL without statin treatment, and only included patients with well-controlled dyslipidemia. Fourth, we only included patients who were treated using EES. This study indicated that the physicians should use EES in this study. However, six patients were treated with biolimus-eluting stents by operator’s discretion because of lesion length, size and location. Biolimus-eluting stent is quite different from EES in regard to stent platform, strut thickness, loaded drug, and polymer. Therefore, in order to avoid the impact of difference between the two stents, we excluded any other second-generation DES. Although EES are more effective and safe coronary stent than first generation DES, there are DES-specific differences among patients with diabetes [39]. Therefore, future studies should examine the potential effects of the drug, polymer, and stent platform. Fifth, most patients with DM were supposed to be in the early stage of DM in this study. There were no patients with severe DM, including insulin user. A prospective study is warranted to evaluate whether glucose fluctuation will have the effect of vessel healing after stenting and late clinical events in patients with severe DM. Finally, to date, direct causal relationship between variability in NIT and clinical endpoint has not been proven in patients with coronary stents. We used an OCT-based 3-D method to quantitatively evaluate the variability in neointimal distribution after stenting to investigate the uniformity of neointima proliferation. Using this method, large glucose fluctuation was associated with higher variability in neointimal thickness within the stent, suggesting more homogeneous neointimal suppression by the control of glucose fluctuation. A sufficiently powered clinical study should be performed to more adequately address the true impact of variability in NIT on future clinical events.

## Conclusions

Glucose fluctuations may affect vessel healing after EES implantation in patients with CAD who are receiving lipid-lowering therapy. Therefore, glucose fluctuations may be an important target for secondary prevention after coronary stenting, which is independent of dyslipidemia control. Nevertheless, future studies should examine the rationale for the early detection and control of glucose fluctuations, based on the wide-spread use of second-generation DES for patients with CAD.

## Abbreviations

CAD: coronary artery disease
CGM: continuous glucose monitoring
DM: diabetes mellitus
MACE: major adverse cardiac events
MAGE: mean amplitude of glycemic excursion
NIT: neointimal thickness
OCT: optical coherence tomography
PCI: percutaneous coronary intervention
VH-IVUS: virtual histology intravascular ultrasound
